# Towards continuous EIT monitoring for hemorrhagic stroke patients

**DOI:** 10.3389/fphys.2023.1157371

**Published:** 2023-04-05

**Authors:** Taweechai Ouypornkochagorn, Nick Polydorides, Hugh McCann

**Affiliations:** ^1^ Faculty of Engineering, Srinakharinwirot University, Bangkok, Thailand; ^2^ School of Engineering, The University of Edinburgh, Edinburgh, United Kingdom

**Keywords:** electrical impedance tomography, multi-frequency, low-noise, image reconstruction, simulation, stroke

## Abstract

The practical implementation of continuous monitoring of stroke patients by Electrical Impedance Tomography (EIT) is addressed. In a previous paper, we have demonstrated EIT sensitivity to cerebral hemodynamics, using scalp-mounted electrodes, very low-noise measurements, and a novel image reconstruction method. In the present paper, we investigate the potential to adapt that system for clinical application, by using 50% fewer electrodes and by incorporating into the measurement protocol an additional high-frequency measurement to provide an effective reference. Previously published image reconstruction methods for multi-frequency EIT are substantially improved by exploiting the forward calculations enabled by the detailed head model, particularly to make the referencing method more robust and to attempt to remove the effects of modelling error. Images are presented from simulation of a typical hemorrhagic stroke and its growth. These results are encouraging for exploration of the potential clinical benefit of the methodology in long-term monitoring of hemorrhagic stroke.

## 1 Introduction

As a result of stroke, more than 100 million people world-wide live with significant disability, and every year more than 10 million people die prematurely (Feigin et al., 2022). To treat the condition soon after onset in any individual patient, a critical requirement is to determine urgently whether the stroke is ischemic (due to a blood clot) or hemorrhagic (due to a bleed). Currently, robust initial diagnosis of stroke requires neuroimaging, typically including X-ray computed tomography and magnetic resonance imaging. Due to resource limitations, it has been estimated (Goren et al., 2018) that such imaging and treatment is applied appropriately in as few as 4% of cases. Although the clot or bleed condition is relatively static for extended periods (Donnan et al., 2008), crucial changes do occur over a range of timescales. Also, it is important to determine the impact of treatment. Therefore, close monitoring of the condition is desirable over periods of hours and days, with adequate temporal resolution to initiate further treatment and to assess potential outcomes. Electrical Impedance Tomography (EIT) may present an opportunity for improved diagnosis and monitoring of stroke, as discussed by a number of authors, e.g., (Brown, 2003) (Holder, 2005) (Adler and Boyle, 2017) (Goren et al., 2018).

In a recent paper (Ouypornkochagorn et al., 2022a), we demonstrated imaging of cerebral hemodynamics on human volunteers by low-noise EIT [signal-to-noise ratio (SNR) from 77 to 95 dB] with 32 scalp-mounted electrodes, using 10 kHz sinusoidal current excitation. The 3-dimensional images obtained by that system displayed cerebral hemodynamic phenomena over timescales ranging from around 20 ms to tens of seconds. For the case of 10 kHz measurement, with simulated 20 mm-diameter “blood” inclusions, it was shown that very low-noise measurements are essential, i.e., with SNR substantially greater than 50 dB. In a subsequent paper (Ouypornkochagorn et al., 2022b), we started discussion of its potential use for stroke monitoring. This is a very challenging application of EIT, for several reasons, including the need to apply a large number of electrodes reliably in a pressurized clinical setting, the absence of reference measurements on the patient prior to stroke onset, and the complexity of the image reconstruction process.

In the present paper, we assess the prospects to simplify the system: firstly, two changes of the measurement protocol are considered, *viz.* Reduction of the number of scalp electrodes to 16, and introduction of reference measurements at 100 kHz; secondly, in the image reconstruction process we consider various possible improvements, relative to previous best practice, to exploit fully the reference measurements. All of the results reported in this paper are deduced from extensive simulations.

Having previously made EIT measurements with more than 50 human volunteers, our experience of applying 32 electrodes on the scalp (plus a reference electrode) in each case, is that extreme care has to be taken to monitor the valid electrical contact of each electrode with the scalp. Nevertheless, our results presented in (Ouypornkochagorn et al., 2022a) suggest that robust localization of cerebral phenomena is achieved. Shi et al. (2018) reported 2-D EIT of the human head with 16 scalp-mounted electrodes and 50 kHz current excitation, achieving 1 fps image rate. In the present paper, we arrange 16 electrodes (see Section 2.1) so as to enable 3-D EIT imaging, and the measurement protocol is such that the relatively high image rate we reported in (Ouypornkochagorn et al., 2022a) is maintained, i.e., 100 fps.

In most previous implementations of EIT, the requirement for a reference measurement has been a crucial limitation. Therefore, it is difficult to apply for stroke localization where there are no measurement data from the time before the stroke occurred. Frequency difference EIT (fdEIT) exploits the case where there is a frequency dependent change in tissue conductivity, and has been discussed by various authors, e.g., (Adler and Boyle, 2017). The simplest fdEIT method images the conductivity change at one frequency relative to that at the reference frequency, using voltage measurements at both frequencies obtained simultaneously, and without the need for a resting-state reference (Packham et al., 2012). The high-frequency voltage measurements are generally used as the reference since the tissue conductivity is usually high and there are small differences in conductivity among the different tissues. This paper follows that approach, using a reference frequency of 100 kHz for current injection in addition to the 10 kHz measurements used in our previous work. This simplest form of fdEIT directly uses the difference vector between the two voltage measurements, which suits a homogeneous and frequency-invariant background. The so-called weighted-fdEIT method improves further on fdEIT by creating a voltage difference vector where the high-frequency voltage vector is scaled by a factor as described in Section 2. Weighted-fdEIT has been widely used since it is more tolerant of variable backgrounds. However, it may not properly reconstruct if the region of conductivity change is large (Jun et al., 2009).

In the case of simulated stroke measurements, the fdEIT technique has been discussed in (Malone et al., 2015). The approach has been elaborated further in (McDermott et al., 2020a) using simulations and the human stroke research dataset provided by (Goren et al., 2018). The latter were gathered by an EIT system comprising 32 electrodes, with measurements made at 17 frequencies in the range 5 Hz–2 kHz, with SNR approximately 45–50 dB in human tests (McDermott et al., 2020a). Whilst some success is reported in (McDermott et al., 2020a) with classification techniques applied to human data without carrying out image reconstruction, and images are also reconstructed from simulated measurements, no reconstructed images have been published to date for human data recorded from stroke patients, to the best of our knowledge.

In the present paper, the focus of our data analysis remains firmly directed at localization of the stroke site. Section 2.2 discusses previously published variations of the fdEIT method for image reconstruction, and our proposed improvements on those. The analysis presented here is restricted to simulation of the hemorrhagic case only. Blood inclusions of volume 50 mL were simulated in (McDermott et al., 2020a) in the hemorrhagic case. In both (Ouypornkochagorn et al., 2022a) and (Ouypornkochagorn et al., 2022b) blood inclusions of 20 mm diameter (i.e., 4.2 mL volume) were simulated. In the present paper, blood inclusion diameters ranging from 20 to 60 mm (i.e., 4.2 mL–113.1 mL in volume) are simulated.

## 2 Materials and methods

### 2.1 Simulated measurement protocol

**FIGURE 1:**
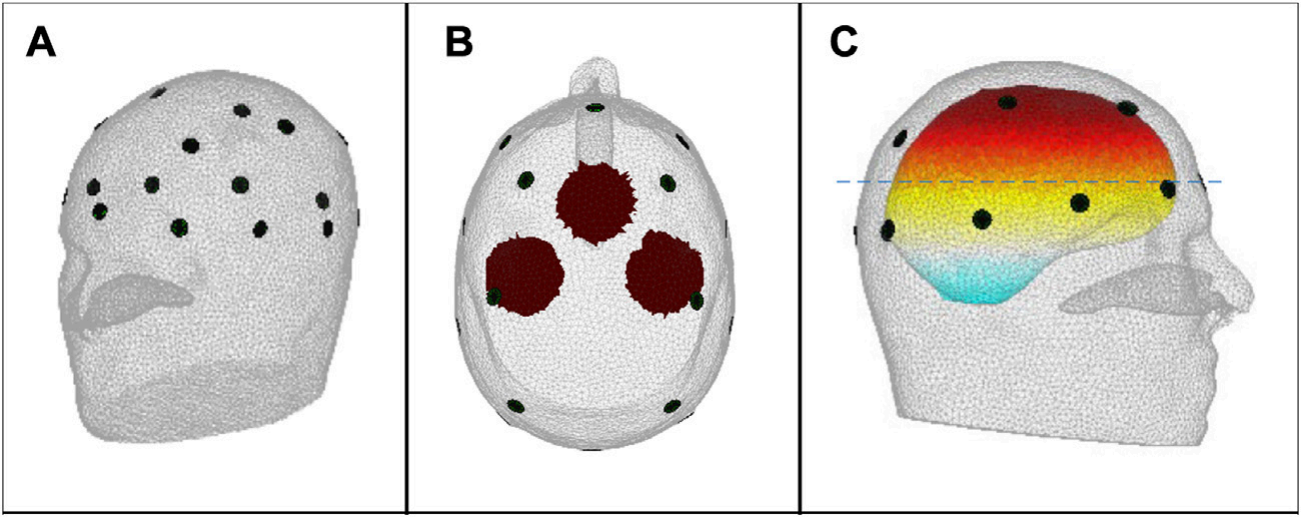
**(A)** The 16-electrode array; **(B)** Top view of the 2-D projection of the simulated 40 mm-diameter spherical blood inclusions, defining the inclusion sites FRONT, LEFT and RIGHT, as used in the text and in Figure 3; Figure 4; and **(C)** Side view of the head, showing the location of the 2-D plane (dashed line) that contains the centre point of the FRONT inclusion; the centre points of the LEFT and RIGHT inclusions lie in a parallel plane that is 10 mm higher in the head.

**FIGURE 2:**
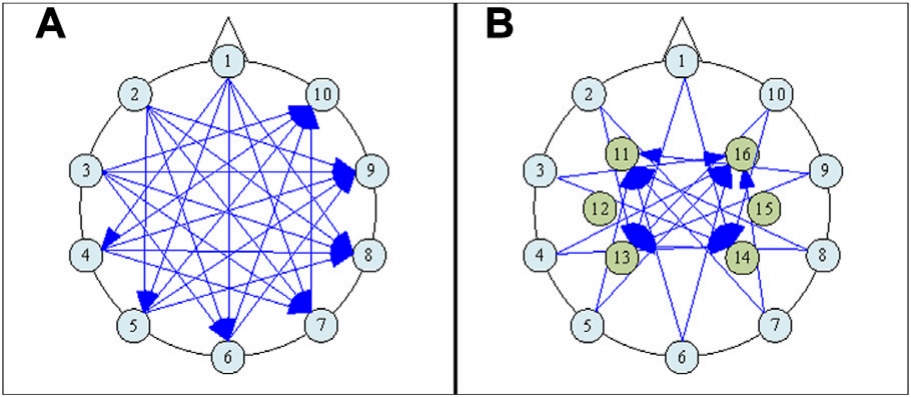
The electrode numbering scheme, **(A)** showing only the plane of 10 and the applied Current Patterns (CPs) involving electrodes only within that plane, and **(B)** including both the plane of 6 and the plane of 10, and showing also the CPs that involve any electrode within the plane of 6.

Figure 1A shows the scalp electrode array containing 16 electrodes of diameter 10 mm based on the international EEG 10–20 system, as included in both forward and inverse models, arranged in a plane of 10 and a plane of 6, with numbering as shown in Figure 2. The contact impedance of the electrodes was set to 1200Ω. Spherical blood inclusions of 40 mm diameter were simulated at three locations: the front, the left, and the right, in the brain region (Figure 1B). In all cases, only one inclusion was simulated. To image the development of a hemorrhage, further blood inclusions of 20 mm, 30 mm, 50 mm, and 60 mm-diameter were simulated at the left side, all at the same center location as the 40 mm case.

Due to the decreased number of sensing electrodes from that used in (Ouypornkochagorn et al., 2022a), we sought to improve measurement distinguishability by following the approach discussed in (Adler et al., 2011), where guidance is offered regarding the optimal arrangement of both current drive and voltage measurement geometries. Accordingly, forty-five opposite-drive current patterns (CPs) were selected, as shown in Figure 2, and 16 electrode measurement pairs (MPs) were assigned, as listed in Table 1, each with approximately 90° electrode separation [the latter condition having been arrived at by considering the risk of voltage measurement saturation in the hardware system described in (Ouypornkochagorn et al., 2022a)]. The current amplitude was 1mA_rms_. Voltage measurements were not simulated for any pair that includes a current-drive electrode, leaving 540 measurement configurations at any frequency.

**TABLE 1:** Measurement pairs (MPs).

MP	Sites	MP	Sites	MP	Sites	MP	Sites
1	1–3	5	5–7	9	9–1	13	13–15
2	2–4	6	6–8	10	10–2	14	14–16
3	3–5	7	7–9	11	11–13	15	15–11
4	4–6	8	8–10	12	12–14	16	16–12

### 2.2 Image reconstruction

The boundary voltages V can be computed from the discretization model function U as in (1), where *ε* is the modelling error and *e*_*m*_ is the combined effect of measurement error and noise. The conductivity σ is frequency-dependent, which results in different values of V at different frequencies ω. The fdEIT method utilizes boundary measurement voltage vectors *V*_*ω1*_ and *V*_*ω2*_ at frequencies ω_1_ (termed “the reference frequency”) and ω_2_ (the “measurement frequency”), to reconstruct the image of the conductivity difference distribution ( $\hat{\sigma }$ ) between the two frequencies. For the simple fdEIT, *V*_*ω2*_ can be directly computed based on *V*_*ω1*_ as in (2), where *J* is the sensitivity matrix (Jacobian matrix) of *σ*(*ω*_*1*_), Δ*σ*_*ω2,ω1*_ is the vector of conductivity difference between the two frequencies, *e*_*L*_ is the linearization error, and Δ*ε* and Δ*e*_*m*_ are, respectively, the remaining modelling error and measurement error of using the conductivity difference. The image of conductivity distribution at frequency *ω*_*2*_ relative to that at *ω*_*1*_ can be estimated by (3). The difference voltage is given by (4).

$$V=U\left(\sigma \left(\omega \right)\right)+\varepsilon \left(\sigma \left(\omega \right)\right)+e_{m}$$ (1)

$$V_{\omega_{2}}=V_{\omega_{1}}+J\left(\sigma \left(\omega_{1}\right)\right)\Delta \sigma_{\omega_{2},\omega_{1}}+e_{L}+\Delta \varepsilon +\Delta e_{m}$$ (2)

$$\hat{\sigma }=\underset{\sigma }{\mathrm{argmin}}\left\{{\left\|V_{\omega_{2}}-U\left(\sigma \right)\right\|}^{2}\right\}$$ (3)

$$\Delta V=V_{\omega_{2}}-V_{\omega_{1}}$$ (4)

Weighted-fdEIT (WfdEIT) is an improved version of the method. Instead of using *V*_*ω1*_ as the reference, a new reference is generated by projecting *V*_*ω2*_ to *V*_*ω1*_ *via* the scalar product of the two, normalized by the magnitude of *V*_*ω1*_, as shown in (5) (Packham et al., 2012) (Jun et al., 2009). Then *αV*_*ω1*_ is used as the reference since it contains the part of *V*_*ω2*_ that behaves in the same way as *V*_*ω1*_ and then Δ*V* is the part of *V*_*ω2*_ that is orthogonal to *V*_*ω1*_. This projection can improve the performance of estimation since Δ*V* contains the information relating to the different behaviors of *V*_*ω2*_ and *V*_*ω1*_ which in turn depend non-linearly upon the different conductivity distributions.

$$\Delta V=V_{\omega_{2}}-\alpha V_{\omega_{1}},where\;\alpha =\left(V_{\omega_{2}}\cdot V_{\omega_{1}}\right)/{\left\|V_{\omega_{1}}\right\|}^{2}$$ (5)

In the situation of head EIT which is severely ill-conditioned and introduces unavoidable modelling error, the estimation becomes more difficult. Model function *U* as in (3) then may not result in an accurate value of *V* at any frequency. Also, inaccurate guesses of initial conductivities worsen the estimation performance further. The conductivities used in the estimation are usually selected from a set of nominal values (*σ*_*N*_) of tissue types from literature review. Each tissue is considered to have homogeneous conductivity.

In this work, we present two methods to improve the fdEIT method. The first one is named “reference-weighted fdEIT” (RWfdEIT). Measurement voltages *V*_*ω1*_ at the reference frequency may be largely inconsistent with the voltage *V*_*ω1N*_ obtained from a model based on *σ*_*N*_, due to the modelling error, *ε*. Therefore, we compute *V*_*ω1N*_ as given by (6) (neglecting measurement error and noise), and use it as the reference. *V*_*ω1*_ is now projected to *V*_*ω1N*_ as shown in (7). *V*_*ω1N*_ is based on *σ*_*ω1N*_ which is the nominal conductivity at *ω*_*1*_, obtained from literature. With RWfdEIT, even though the modelling error is distinctly added to the estimation [with *αε* in (7)], this results in the same basis being applied to both the model function and the estimation reference.

$$V_{\omega_{1N}}=U\left(\sigma_{\omega_{1N}}\right)=V_{\omega_{1}}+\varepsilon$$ (6)

$$\Delta V=V_{\omega_{2}}-\alpha V_{\omega_{1N}},where\;\alpha =\left(V_{\omega_{1}}\cdot V_{\omega_{1N}}\right)/{\left\|V_{\omega_{1N}}\right\|}^{2}$$ (7)

The second proposed new method is an improvement of the RWfdEIT by applying the approach adopted in (Ouypornkochagorn et al., 2022a). If *σ*_*ω1N*_ is close to *σ*_*ω1*_, the estimation can be carried out with the addition of Jacobian error *e*_*J*_, (8) and (9). The conductivity estimation ${\tilde{\sigma }}_{\omega_{2}}$ then may be slightly different to the expected *σ*_*ω2*_ when the used model is close to the exact geometry.

$$J\left(\sigma \left(\omega_{1}\right)\right)\Delta \sigma_{\omega_{2},\omega_{1}}\approx J\left(\sigma_{\omega_{1N}}\right)\Delta {\tilde{\sigma }}_{\omega_{2},\omega_{1N}}\;;\Delta {\tilde{\sigma }}_{\omega_{2},\omega_{1N}}={\tilde{\sigma }}_{\omega_{2}}-\sigma_{\omega_{1N}}$$ (8)

$$J\left(\sigma \left(\omega_{1}\right)\right)\Delta \sigma_{\omega_{2},\omega_{1}}=U\left({\tilde{\sigma }}_{\omega_{2}}\right)-U\left(\sigma_{\omega_{1N}}\right)+e_{J}$$ (9)

When (9) is substituted into (2) to give (10), it can then be rewritten as (11). The new variable ${\tilde{V}}_{\omega_{2}}$ is now the substitution of *V*_*ω2*_ where *U*(*σ*_*ω1N*_) and *V*_*ω1*_ are already known. The image of conductivity distribution is now computed with (12) which is a similar form to (3), and since the computation is based on *σ*_*ω1N*_, the difference image is obtained from (13). Since this method is a modification of RWfdEIT which is based on the simple fdEIT method, the projection of *V*_*ω1*_ is then applied. The voltage difference vector between the two frequencies is still as given in (7).

$$V_{\omega_{2}}=V_{\omega_{1}}+U\left({\tilde{\sigma }}_{\omega_{2}}\right)-U\left(\sigma_{\omega_{1N}}\right)+\eta \;where\;\eta =e_{J}+e_{L}+\Delta \varepsilon +\Delta e_{m}$$ (10)

$${\tilde{V}}_{\omega_{2}}=U\left({\tilde{\sigma }}_{\omega_{2}}\right)+\eta \;where\;{\tilde{V}}_{\omega_{2}}=V_{\omega_{2}}-V_{\omega_{1}}+U\left(\sigma_{\omega_{1N}}\right)$$ (11)

$$\tilde{\sigma }=\underset{\tilde{\sigma }}{\mathrm{argmin}}\left\{{\left\|{\tilde{V}}_{\omega_{2}}-U\left(\tilde{\sigma }\right)\right\|}^{2}\right\}$$ (12)

$$\Delta \hat{\sigma }=\tilde{\sigma }-\sigma_{\omega_{1N}}$$ (13)

The main improvement of this proposed method is noticeable from (10). Compared with (1), the modelling error and noise are expected to be reduced, respectively, to Δ*ε* and Δ*e*_*m*_. However, the difference between the exact geometry and that used in the computation model should not be too large, since the selection of *σ*_*ω1N*_ must be close to *σ*_*ω1*_ in order to avoid large values of Jacobian error and linearization error, *e*_*J*_ and *e*_*L*_. We name this method “reference-weighted fdEIT with modelling error deduction” (RWfdEIT-med). *V*_*ω2*_ is modified to ${\tilde{V}}_{\omega_{2}}$ with the use of *V*_*ω1N*_. The reference is now *U* (*σ*_*ω1N*_) that allows iterative non-linear estimation to be easily implemented.

Image reconstructions in this study were performed by using fdEIT, WfdEIT, RWfdEIT, and RWfdEIT-med. The solution procedure used was that presented at length in (Ouypornkochagorn et al., 2022a), based on the Regularized Newton-Krylov Generalized Minimal Residual (GMRes) method with 200-long Krylov subspaces and smoothness prior implemented as in (14). The regularization parameter was set to 1 × 10^-8^ (rather than 1 × 10^-7^). For the first three methods, the image results obtained by the single-step solution were much superior to those obtained by iteration, and only the single-step results are presented in Section 3. For the RWfdEIT-med algorithm, the number of iterations was 10 [rather than 15 as in (Ouypornkochagorn et al., 2022a)] which sufficed to make the solution converge. The conductivity change over the skull region was fixed during the estimation. EIDORS software (Polydorides and Lionheart, 2002) was used for forward computation.

$$\Delta \tilde{\sigma }={\left(J^{T}J+\lambda R^{T}R\right)}^{-1}J^{T}\left(V_{\omega_{2}}-U\left(\sigma \right)\right)$$ (14)

### 2.3 Simulation models for stroke localization

Two head models, the forward model and the inverse model, were constructed [both models based on the 53k model provided by the UCL group of Prof. D. Holder (Gilad et al., 2007)]. The forward model has 227k elements. Despite having five tissues in the forward model geometry (i.e., scalp, skull, cerebrospinal fluid, gray and white matter), only three conductivity values were implemented, i.e., for scalp, skull, and a single common value (the “brain” value) for the remaining three tissues. The tissue conductivities at each frequency are shown in Table 2. The inverse model geometry, with 134k elements and only three tissue types, was designed to be less complicated than the forward model, hence significant modelling error is expected. For the 40 mm-diameter inclusions at positions FRONT, LEFT and RIGHT shown in Figure 1B, simulated measurement datasets were calculated for the noise-free case and then with the addition of noise for two different noise conditions, *viz.*70 dB and and 50 dB SNR. Ten repeats of measurement simulation were performed for each SNR, and the data from each simulation were input to all four reconstruction methods. To image the growth of a hemorrhage, only the 70 dB SNR case was simulated.

**TABLE 2:** Head tissues: Assigned conductivity values.

Tissue type	Conductivity @ 10 kHz (S/m)	Conductivity @ 100 kHz (S/m)
Scalp	0.25 Yamamoto and Yamamoto. (1976)	0.40 Yamamoto and Yamamoto. (1976)
Skull	0.0125 Tang et al. (2008)	0.0133 Tang et al. (2008)
Brain	0.2556 Latikka et al. (2001)	0.2770 Latikka et al. (2001)
Blood inclusion	0.6452 Anas et al. (2012)	0.771 Faes et al. (1999)
	Faes et al. (1999)	

To obtain accurate localization performance, the reconstructed tetrahedral images were first transformed into cubic voxel images with an equal size of 1.5x1.5x1.5 mm^3^, yielding a total of 2,026,944 voxel elements, each of volume 3.375 mm^3^ *.* Since the conductivity change due to a blood inclusion is positive, it is desirable to show images of only those elements in the reconstructed images that exhibit a positive change in conductivity. Following the method of (Yasin et al., 2011), the maximum sum of voxel values along the x-y plane, the x-z plane, and the y-z plane of the image were used to determine the center of gravity (CoG) of the reconstructed blood inclusion (*cog*_*recons*_). The localization error (LE) is then determined from (15) where *c*_*target*_ is the true center of the inclusion.

$$LE=\left\|c_{target}-cog_{recons}\right\|$$ (15)

## 3 Results

**FIGURE 3:**
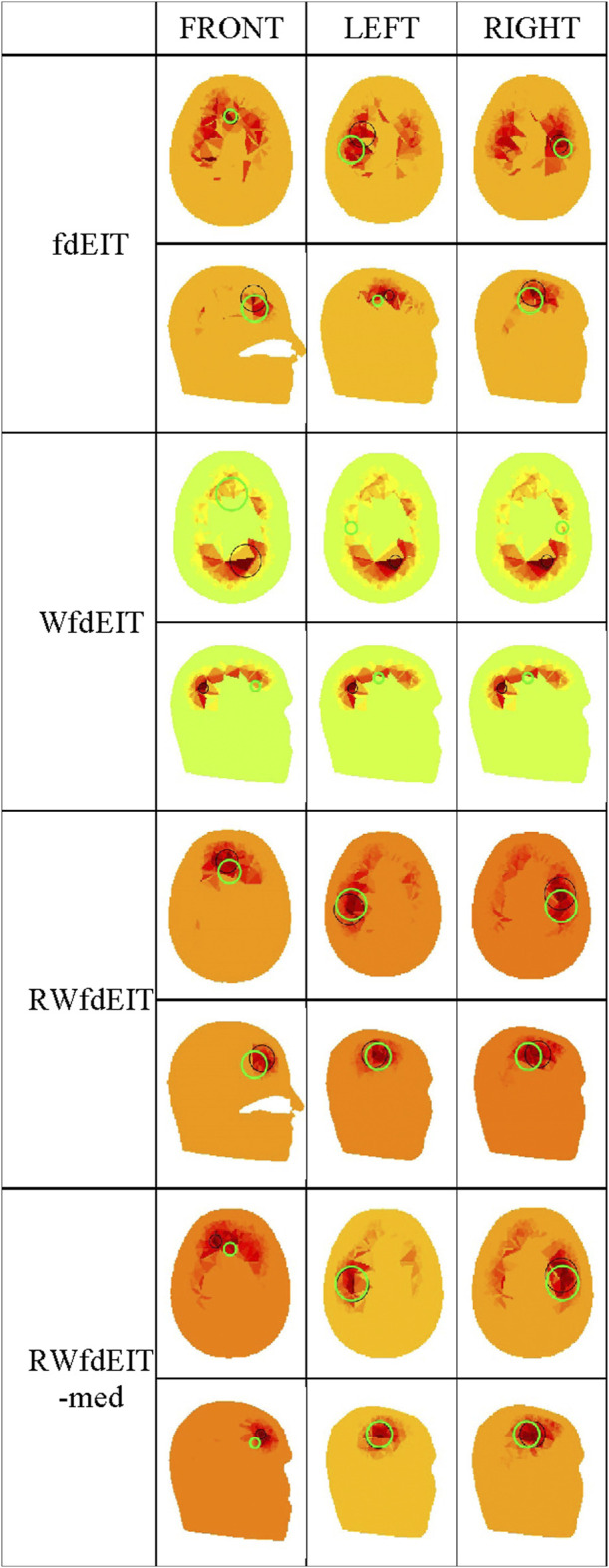
Noise-free case: reconstructed images of conductivity change due to the inclusions shown in Figure 1B, for each reconstruction method (as given in the leftmost column), sectioned at the CoG (axial and sagittal views). Green circles show the location of the true simulated inclusion within the CoG section, and black circles show the location of the reconstructed object (with diameter equal to that of the true sphere section).

**FIGURE 4:**
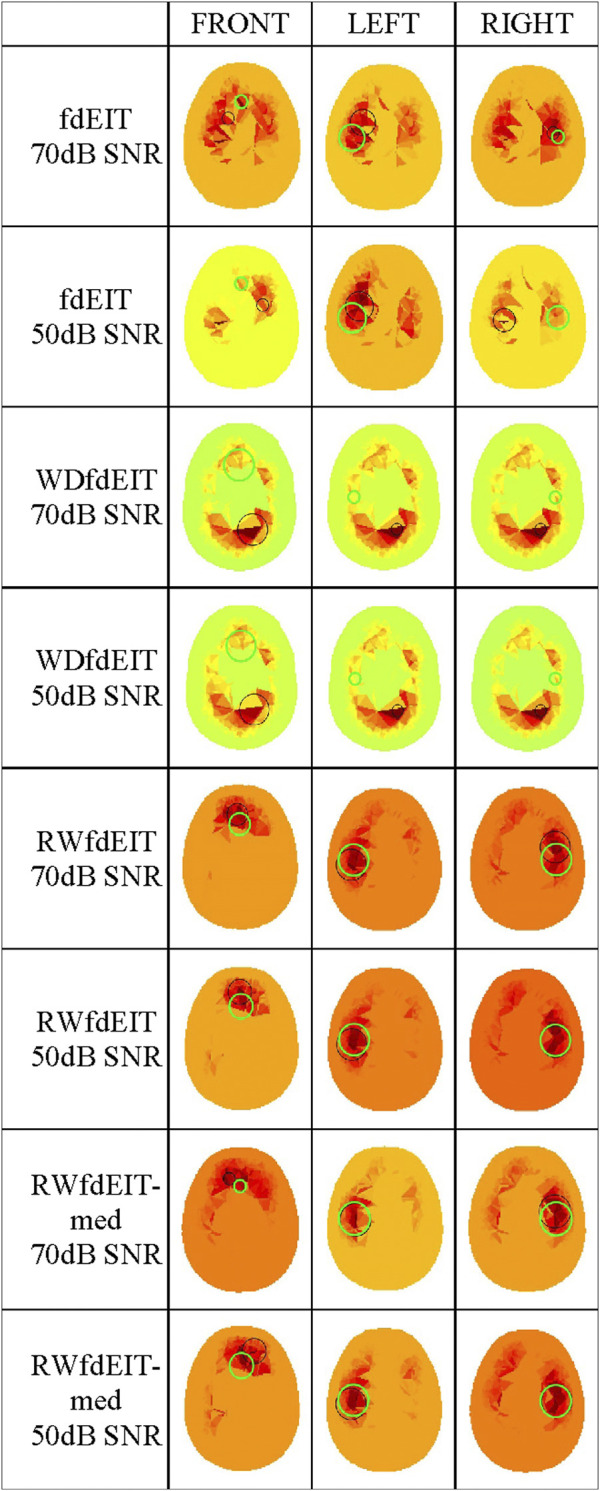
Noise cases 70 dB and 50 dB SNR: reconstructed images of conductivity change due to the inclusions shown in Figure 1B, for each reconstruction method (as given in the leftmost column, with the SNR value), sectioned at the CoG (axial view). Green and black circles are as described in Figure 3.

For the 40 mm-diameter blood inclusions at the three different locations FRONT, LEFT and RIGHT, images yielded by the various reconstruction methods in the noise-free case are shown in Figure 3, and corresponding images in both noise cases are shown in Figure 4. The mean localization errors (and their standard deviation) for the reconstructed blood inclusions are given in Tables 3–5. (A standard deviation with a value of zero arises when all repeat simulations return distributions that are almost identical in spatial terms, indicated in the Tables below by *, or when the variation of CoG position among all simulations is less than the 1.5 mm grid size, indicated by **. In both cases, very small variations of the magnitude of conductivity change are observed between the images.)

**TABLE 3:** Localization Error (mm) of the front blood inclusion.

SNR	fdEIT	WFdEIT	RWfdEIT	RWfdEIT-med
∞	16.03	82.32	16.38	24.66
70 dB	25.87 ± 8.5	82.32*	17.73 ± 2.1	24.36 ± 0.9
50 dB	31.07 ± 11.6	76.78 ± 16.3	19.89 ± 3.5	23.47 ± 6.8

(Mean ± Standard Deviation, except in the no-noise case. * See text.).

Considering first of all the fdEIT image reconstruction method, there are large positive artefacts on the left and the right side for all blood inclusion positions, meaning that the recovered inclusion position is not distinct. In the noise-free case, the fdEIT method yields mean localization errors varying from 13 mm to 24 mm (illustrated in Figure 3), some of which are worsened substantially when noise is added in the simulation (Figure 4). For the 50 dB SNR condition the reconstructed images are extremely inconsistent from one simulation to the next in most cases.

The simplest version of the weighted fdEIT reconstruction method (WfdEIT) always returns a nearly-identical distribution (see Figure 3; Figure 4), with very large localization errors in all cases (see Tables 4, 5). In the case of 70 dB SNR noise, the returned value of the standard deviation of the mean LE is identically zero in some cases in Tables 4–6, as discussed above. Considering all of these factors, this method is deemed to have failed.

**TABLE 4:** Localization Error (mm) of the left blood inclusion.

SNR	fdEIT	WFdEIT	RWfdEIT	RWfdEIT-med
∞	24.25	71.67	7.60	2.51
70 dB	23.12 ± 1.5	71.67*	7.60**	3.35 ± 1.8
50 dB	23.40 ± 6.4	69.87 ± 1.7	6.36 ± 3.1	5.63 ± 2.6

(Mean ± Standard Deviation, except in the no-noise case. * and ** See text.).

**TABLE 5:** Localization Error (mm) of the right blood inclusion.

SNR	fdEIT	WFdEIT	RWfdEIT	RWfdEIT-med
∞	13.00	47.06	15.56	9.15
70 dB	13.95 ± 1.2	47.06*	16.14 ± 0.6	9.15**
50 dB	21.43 ± 16.3	43.76 ± 9.5	11.36 ± 6.4	10.70 ± 6.3

(Mean ± Standard Deviation, except in the no-noise case. * and ** See text.).

**TABLE 6:** Localization Error (mm) of the left blood inclusion—Noise free case.

Diameter (mm)	fdEIT	RWfdEIT	RWfdEIT-med
20	44.89	62.40	13.65
30	22.11	6.04	5.20
40	24.25	7.60	2.51
50	13.44	8.07	1.80
60	13.44	8.07	1.80

When the proposed new reference-weighted methods were applied (RWfdEIT and RWfdEIT-med), the imaging performance was improved significantly. The blood inclusion is clearly observable at all sites, as shown in Figure 3 (noise-free case) and Figure 4 (70 dB and 50 dB SNR). For all inclusion sites and all noise conditions, the localization error is substantially smaller than the 40 mm diameter of the simulated blood inclusion. The mean localization error in each simulated case shows a very strong consistency between the noise-free and noise-added conditions. Localization error is seen to vary substantially between the three inclusion sites.

When noise is added, the noise-tolerant behavior of these algorithms sometimes results in zero values of standard deviation of LE due to the CoG positions of the (non-identical) conductivity distributions being separated by less than the 1.5 mm image resolution. In general, adding noise into the simulations had only minor impact on the performance of both methods. Particularly, the performance in the 70 dB SNR case was about the same as in the noise-free case. For the FRONT site, the images produced by the RWfdEIT method have higher image amplitude than those from the RWfdEIT-med method, but for the LEFT and RIGHT sites, those produced by the RWfdEIT-med method have larger amplitude.

**FIGURE 5:**
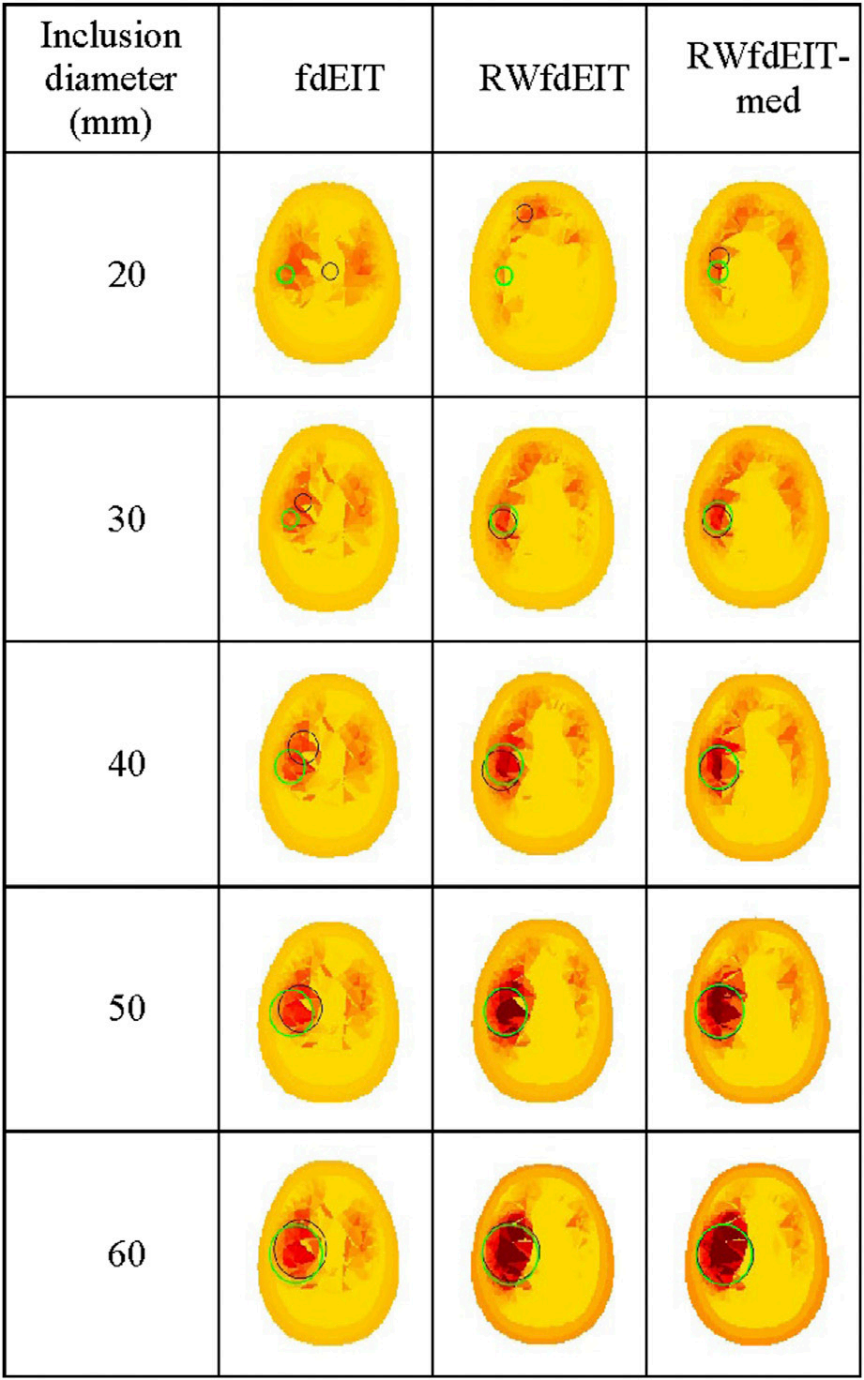
Reconstructed images of conductivity change due to an inclusion growing from 20 mm- to 60 mm-diameter at the LEFT site shown in Figure 1B, for the 70 dB SNR noise case, sectioned at the CoG (axial view). The reconstruction method is given at the top of each column of images. Green and black circles are as described in Figure 3.

The algorithms fdEIT, RWfdEIT and RWfdEIT-med were used to reconstruct images for simulated blood inclusions varying in size from 20 mm to 60 mm diameter, at the LEFT site, in noise-free and 70 dB SNR noise conditions. Table 6 shows the localization errors obtained in the noise-free condition, where the RWfdEIT-med method above shows markedly superior performance. Figure 5 shows all three reconstructed images for one example simulation, at each inclusion diameter. When the inclusion diameter is 20 mm, the fdEIT and RWfdEIT methods did not locate the inclusion successfully, whereas it was located successfully using the RWfdEIT-med algorithm.

**FIGURE 6:**
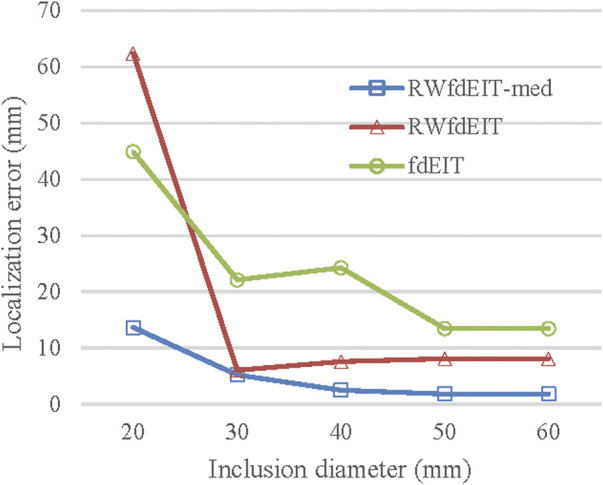
Localization error (noise-free case) for blood inclusion size varying from 20 mm- to 60 mm-diameter.

When the inclusion was increased in size, the localization performance of all three methods improved, as shown in Figure 6. The standard deviation of LE in each set of reconstructions is much smaller than the LE value. However, the fdEIT and RWfdEIT methods tended to return identical distributions for the larger inclusion sizes. In general, the presence of small noise levels (70 dB SNR) caused only slightly change of the localization error.

## 4 Discussion

This study is strongly focused on development of the various hardware and computation methods used in the so-called fEITER system (Ouypornkochagorn et al., 2022a) to address the problem of hemorrhagic stroke; hence, the choice of 10 kHz as one of the two measurement frequencies. A desire to retain the excellent temporal resolution of fEITER mandated that the second frequency should be significantly larger than 10 kHz. However, the scarcity of reliable *in vivo* measurements of the electrical properties of tissues and fluids in the head, and their frequency dependence, is a major concern. The references cited in Table 2 are representative of the available literature and it is hoped that further measurements will become available in future. Meanwhile, there is a great deal of scope to apply the methods presented in this paper to different frequency pairs.

As with the 20 mm-diameter inclusions simulated in (Ouypornkochagorn et al., 2022a), the present simulation study achieves (with RWfdEIT-med reconstruction) good localization of inclusions of that size and larger, even with only half the number of electrodes, and in spite of the complexity of carrying out frequency-difference EIT.

Bearing in mind the strong focus of the present study, as noted above, it cannot be considered to be a general appraisal of all four image reconstruction methods fdEIT, WfdEIT, RWfdEIT and RWfdEIT-med for frequency-difference EIT. Nevertheless, localization with the fdEIT and WfdEIT methods has been shown here to be unsuitable for hemorrhagic stroke monitoring even in the situation that the head geometry of the inverse and the forward model is about the same. Compared to single-frequency EIT where the conductivity change is usually small, the conductivity change due to the frequency change in the multi-frequency EIT case is much larger, causing the condition of the system matrix to become crucial. The difference between the measurement voltage and the computed voltage is also usually large for head EIT. For example, from simulations in this study, the inverse voltage has 31% norm error compared with that of the forward model even using the same electrode and conductivity configuration. This means that it is difficult for the WdfEIT method to obtain a good reference by projection, leading to the failed localization performance.

Both of the proposed new reference-weighted methods perform substantially better, as illustrated in Figure 3; Figure 4; Figure 5. In an attempt to overcome modelling error, both reference-weighted methods make use of the detailed head model: RWfdEIT projects the voltage data measured at the reference frequency (100 kHz) onto that calculated from the model function, and therefore the reference data is now in the same basis as the model function; the RWfdEIT-med algorithm also changes the measurement data at the lower frequency (10 kHz) to be in the same basis as the model function and the discussion in Section 2.2 suggests that this reduces the influence of both modelling and measurement errors. Both of the new methods were successful in localization. In general, the performance of the RWfdEIT-med method is found to be slightly better than the RWfdEIT method in terms of localization and image amplitude, which is attributable to the reduction of the modelling error from *ε* to Δ*ε* and the reduction of the measurement error from *e*_*m*_ to Δ*e*_*m*_ as in (10). However, the RWfdEIT-med method introduces the Jacobian error (*e*_*J*_) into the estimation as in (9). This error could be large and region-dependent. Therefore, the localization performance in certain regions then may be poorer than the RWfdEIT method.

In all attempts to carry out iterative image reconstruction in this paper, the Jacobian matrix was updated at each iteration step in order to implement a non-linear process. The success of the iterative process in the RWfdEIT-med method, compared with the very poor results obtained by iteration in the other three methods, and also the much better performance of those three methods by single-step solution (compared to their iterative versions), reflect the need for a very accurate model in the non-linear process and careful handling of discrepancies of model *versus* measurement data.

Clearly the RWfdEIT-med method outperforms the others in the present study and it exhibits a smooth evolution of LE with inclusion size, as shown in Figure 6. No attempt has been made to estimate the size of the inclusion that might be inferred from the reconstructed images, but the amplitude of change in the conductivity distribution, close to the CoG, suggests that it may be fruitful to pursue that aim by appropriate image analysis methods.

Our previous work on human volunteers (Ouypornkochagorn et al., 2022a) has demonstrated the capability of low-noise EIT with scalp-mounted electrodes to image cerebral hemodynamics. In terms of the potential clinical utility of EIT in stroke monitoring, the results shown in the present simulation study (Section 3) are extremely encouraging in that they demonstrate a) successful imaging capability using an EIT system with only 16 scalp-mounted electrodes, provided that measurement noise is as good as in (Ouypornkochagorn et al., 2022a), and b) the success of the multi-frequency approach in providing reference measurements. That no reconstructed images have been published to date for human data recorded from stroke patients, to the best of our knowledge, is most likely attributable to the relatively high noise level in those studies to date; the 32-electrode EIT system used in (Goren et al., 2018), whilst providing the most systematic EIT dataset yet available for human stroke, had measurement SNR approximately 45–50 dB (McDermott et al., 2020a).

For clinical decision-making, methods of EIT data analysis that do not generate reconstructed images have been studied in the multi-frequency case (McDermott et al., 2020a) and the bi-frequency case (McDermott et al., 2020b). The latter concludes that SNR of 60 dB or higher is required if the method is to be feasible.

## 5 Conclusion

The simulation study presented here indicates that a 16-electrode EIT system with the noise properties of the system described in (Ouypornkochagorn et al., 2022a) is adequate for localization and monitoring of hemorrhagic stroke. Its utility is further enhanced by the use of two measurement frequencies in order to provide a suitable reference condition and to exploit the frequency-difference method of EIT. A new method of image reconstruction for multi-frequency EIT is presented and shown to substantially outperform methods reported previously. These results motivate further research towards clinical trial of EIT in stroke monitoring.

## Data Availability

The original contributions presented in the study are included in the article/supplementary materials, further inquiries can be directed to the corresponding author.
